# Bacteriophage-mediated manipulations of microbiota in gastrointestinal diseases

**DOI:** 10.3389/fmicb.2022.1055427

**Published:** 2022-11-17

**Authors:** Lynn El Haddad, Jesus F. Mendoza, Christian Jobin

**Affiliations:** ^1^Department of Medicine, University of Florida, Gainesville, FL, United States; ^2^Department of Molecular Genetics and Microbiology, Gainesville, FL, United States; ^3^Department of Anatomy and Cell Biology, University of Florida, Gainesville, FL, United States; ^4^Department of Infectious Diseases and Immunology, University of Florida, Gainesville, FL, United States

**Keywords:** phages, phageome, bacteriome, gut, inflammation, colorectal cancer

## Abstract

Although some gastrointestinal diseases could be managed using various antibiotics regimen, this therapeutic approach lacks precision and damages the microbiota. Emerging literature suggests that phages may play a key role in restoring the gut microbiome balance and controlling disease progression either with exogenous phage intervention or filtered fecal transplantation or even engineered phages. In this review, we will discuss the current phage applications aiming at controlling the bacterial population and preventing infection, inflammation, and cancer progression in the context of gastrointestinal diseases.

## Introduction

Chronic gastrointestinal (GI) inflammation constitutes the main cause for the development of GI malignancies. Patients with inflammatory bowel disease (IBD) and small intestinal bacterial overgrowth (SIBO) experience a prolonged intestinal inflammation and are exposed to immunosuppressive therapies increasing their risk of developing intestinal malignancies (Stidham and Higgins, 2018). These include colorectal cancer (CRC), small bowel adenocarcinoma, intestinal lymphoma, anal cancer, cholangiocarcinoma as well as extra-intestinal malignancies (Johnson et al., 2013; Axelrad et al., 2016; Stidham and Higgins, 2018). Additionally, chronic GI diseases worsen quality of life, social and work life causing weak GI mobility, dysfunctional gut-brain communication, mucosal immune activation, and gut microbiota alteration (Holtmann et al., 2016; Singh et al., 2020, 2022). Therapies used to treat intestinal inflammation such as corticosteroids and immunosuppressive agents are often prescribed for long durations, focus mostly on the relief of clinical symptoms without targeting the underlying cause, and may promote carcinogenesis (Axelrad et al., 2016; Magro et al., 2020). Additionally, antibiotics lack taxonomic precision and eliminate large consortium of bacteria including beneficial community with anti-inflammatory properties hindering the treatment of CRC patients (Zheng et al., 2019).

The human enteric microbiota is composed of diverse bacteria, fungi, archaea, viruses, and eukaryotic entities. It is considered an essential organ, playing a role in immune homeostasis, host metabolism, and prevention of pathogens colonization (Buffie and Pamer, 2013; Belkaid and Hand, 2014; Gensollen et al., 2016; Rowland et al., 2018). Indeed, the composition of the bacterial community (bacteriome) and the emergence of antibiotic-resistant bacteria have been linked to intestinal malignancies (Gagnière et al., 2016; Simin et al., 2020). Additionally, changes in the bacteriome, including reduced diversity and expansion of specific bacterial taxa can lead to dysregulated immune responses, triggering inflammation such as Crohn’s disease (CD) and Ulcerative colitis (UC), the two main forms of IBD (Chen et al., 2017; Weiss and Hennet, 2017; Hou et al., 2022). Studies showed that IBD patients have a potential different microbial signature than healthy controls with a significant decrease in commensal beneficial bacteria such as *Faecalibacterium* and *Bifidobacterium* and an increase in *Enterobacteriaceae* and *Bacteroides* (Yang and Jobin, 2017; Zamani et al., 2017; Pittayanon et al., 2019; Zhang et al., 2021b). Additionally, the presence of *Fusobacterium nucleatum* in CRC patients has been shown to compete with the beneficial butyrate-producing *Clostridium butyricum* promoting tumor progression and chemotherapy resistance (Yang and Jobin, 2017; Handley and Devkota, 2019; Kannen et al., 2019; Zheng et al., 2019). Other pro-inflammatory and pro-tumoral bacteria such as adherent invasive *Escherichia coli* (AIEC) and *Klebsiella pneumoniae* were found to colonize the gut of individuals with worsening clinical symptoms of inflammation and intestinal tumor progression (Darfeuille-Michaud et al., 1998; Elhenawy et al., 2019; Gogokhia et al., 2019; Pope et al., 2019; Federici et al., 2022).

So far, most research has been focused on the bacteriome signatures in GI diseases and malignancies. However, recent studies have revealed that the viral portion of the microbiome, composed essentially of bacteriophages, i.e., phages, seem to interact with both the bacteriome and the host playing a major role in homeostatic regulation and disease progression in patients with GI inflammation and malignancies (Virgin, 2014; Pfeiffer and Virgin, 2016; Mirzaei and Maurice, 2017; Mukhopadhya et al., 2019). Indeed, research has shown the presence of potential phage signatures or biomarkers in patients with IBD and CRC compared to healthy individuals (Hannigan et al., 2018b; Handley and Devkota, 2019). Here, we review the available data on the role of phages and the presence of potential phage-based signatures between healthy individuals and those with chronic intestinal inflammation and malignancies.

## Bacteriophages

Phages are the most abundant natural entities on planet Earth with an estimate of 10^31^ phage particles (Clokie et al., 2011). Phages can either undergo a lytic or a lysogenic cycle (Kannen et al., 2019). The lytic infection cycle results in the bacterial lysis and the release of phage copies whereas temperate phages integrates into the host bacterial chromosome (prophages) without causing cell lysis, until a certain stimulator initiates the lytic phase resulting in bacterial cell death (Hu et al., 2021). The antimicrobial activity of phages as modulators of microbial communities is assumed by the virulent or lytic phages whereas temperate phages contribute to the pathogenicity and coexistence of the bacteria in the ecosystem (Gogokhia et al., 2019). Of note, genome similarity at the nucleotide and protein level has been proposed to be employed for taxonomic classification of phages compared to the previously used morphology classification (Turner et al., 2021).

The rise of antibiotic resistance is one of the biggest current threats to global health causing increased bacterial infections, mortalities, and financial burden. This crisis calls for an urgency in the development and implementation of a natural therapeutic strategy (Altamirano and Barr, 2019). The prevalence and target-specific nature of phages represent a potential remarkable substitute to outdated antibiotics against emerging pathogens (Khan Mirzaei and Deng, 2022). Phages harbor bactericidal activity and proliferate in a localized fashion to target bacteria. In contrast to antibiotics, phages are able to eradicate their target bacteria preventing a potential bacterial resistance (Altamirano and Barr, 2019). Additionally, their self-limiting infection capacity is critical in maintaining microbiota homeostasis; thus, posing regulatory effects on metabolic activities and overall functional composition at a site. Moreover, no major side effects related to the administration of phages have been documented so far permitting their safe use *in vivo* (Sarker et al., 2016; El Haddad et al., 2018; Gindin et al., 2019; Grubb et al., 2020; Federici et al., 2022). Interestingly, antibiotic-resistance can be resolved through the coupled effects of targeted bacteriophage cocktail treatment with antibiotics (Modi et al., 2013; Schooley et al., 2017).

## Targeted phage therapy

### Phages to control GI disease progression

Research has focused on evaluating the efficacy of phage cocktails in reducing the levels of pathogenic bacteria using *ex vivo* and *in vivo* experiments with murine and human intestinal samples. Gogokhia et al. observed the effect of phages isolated from a patient with IBD on AIEC gut colonization and tumor growth in an *Apc^Min/+^* mouse model, a model of intestinal tumorigenesis (Barker et al., 2009; Gogokhia et al., 2019). The phage mixture, continuously added to the water, was able to reduce AIEC colonization, decrease the tumor size, and protect the mice from an invasive bacteria-causing cancer, thus improving overall mice survival. Additionally, genes associated with cancer initiation, growth, and metastasis were down-regulated in mice that received the AIEC-specific phages. Of note, the addition of phages alone without their bacterial target had no effect on tumor growth (Gogokhia et al., 2019). In another study, Galtier et al. showed that a single dose of a 3 phage-cocktail was able to significantly decrease the number of AIEC in feces and in the intestines of transgenic mice and DSS-induced colitis mice colonized with AIEC over a 14 day-period. The cocktail was also effective in homogenates of ileal biopsies taken from CD patients demonstrating that phages constitutes a viable treatment option against AIEC infections in IBD patients (Galtier et al., 2017). Federici et al. used phage therapy to target a clade of *K. pneumoniae* that was identified in 4 geographically different IBD cohorts (France, Germany, Israel, and the US) and that exhibited a unique resistome and mobilome signature. The authors designed and tested the efficacy of a 5-phage cocktail, orally administered 3 times per week, in reducing *K. pneumoniae* abundance and decreasing colonic inflammation in pre-clinical IBD mice models. It was shown that the phage combination effectively inhibited *K. pneumoniae* loads in the feces and intestinal mucosa and significantly decreased intestinal inflammation. Phages were stable and persisted within the mouse and human simulated GI tract (Federici et al., 2022).

**Figure 1:**
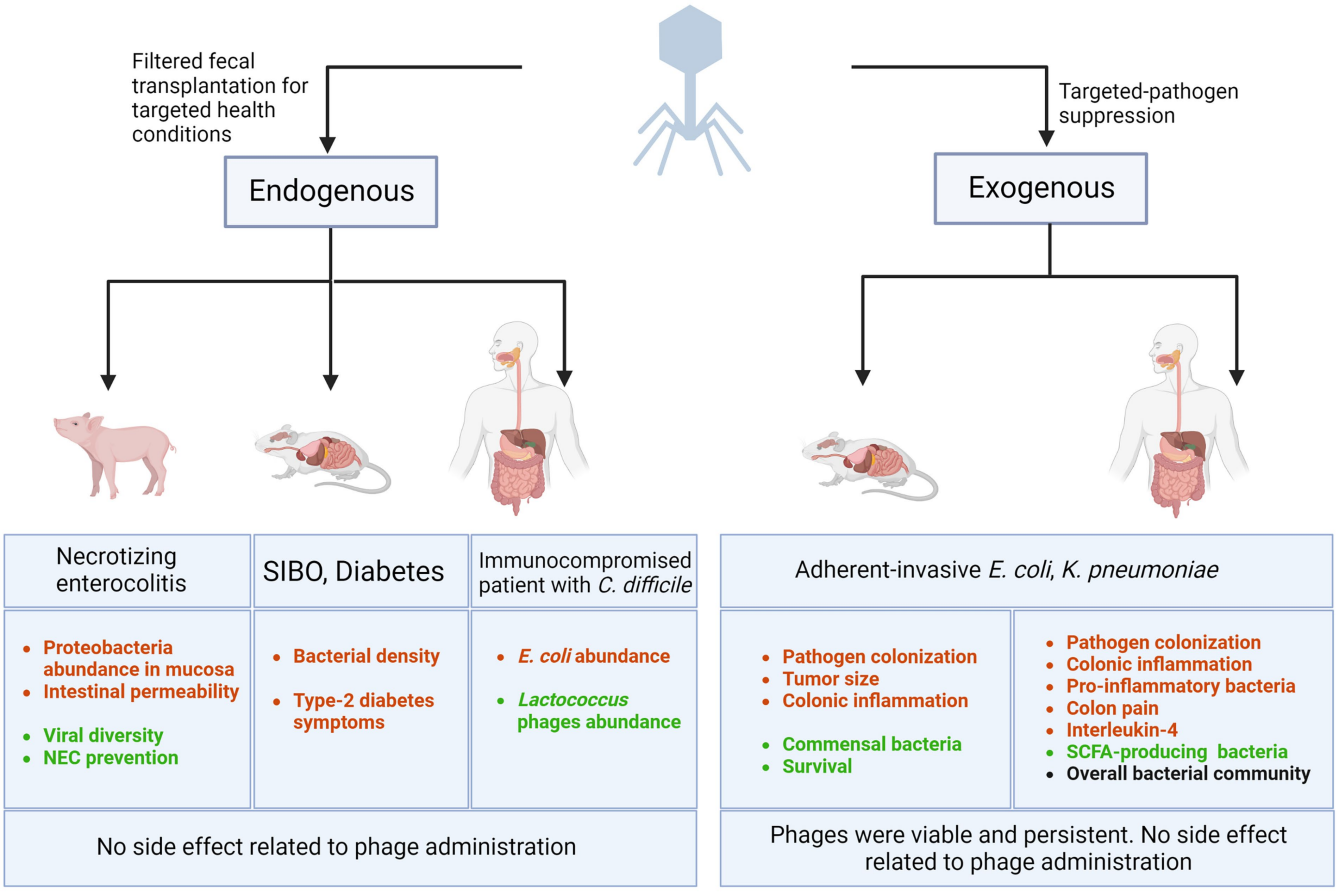
Bacteriophage-related interventions in GI diseases. Lytic phages are being used (1) as the components of filtered fecal matter transplanted in animals and humans (or endogenous phages) to combat health conditions such as necrotizing enterocolitis, Small intestinal bacterial overgrowth (SIBO), diabetes, and recurrent *C. difficile* colonization, (2) as targeted-pathogen suppressor (or exogenous phages) by administering specific phages to animal models or humans to eradicate a selective bacterial pathogen and improve symptoms, survival, and patient outcomes. Decreased outcomes are represented in red and improved outcomes are shown in green. Black text represents consistent and stable outcome. Created with BioRender.com.

Figure 1 summarizes the efficacy and safety of phage addition (exogenous) against specific bacterial pathogens. However, none of these studies investigated whether the phage cocktail modified the microbiota.

Phages have also been used as exogenous agents to change the gut bacteriome composition. It was shown that the administration of phages in mice models can directly impact susceptible intestinal bacteria but also could have a cascading effect on other bacterial species in the gut environment (Bao et al., 2018; Hsu et al., 2018; Lin et al., 2019). Additionally, Zheng et al. targeted the cancer-promoting strain *F. nucleatum* in *Apc^Min/+^* mice, piglets and in clinical patient samples using a phage isolated from human saliva (Zheng et al., 2019). Interestingly, the authors developed dextran (prebiotic) nanoparticles loaded with a CRC drug, irinotecan, and covalently linked this nanoparticle to the phages to construct a phage-guided biotic-abiotic hybrid nanosystem. This phage-guided dextran nanosystem was able to accumulate in cancer tumors eliminating intratumoral *F. nucleatum*, allowing the flourishing of *C. butyricum* and colonic short-chain fatty acids, reversing resistance to chemotherapy, and suppressing the growth of cancer cells (Zheng et al., 2019).

### Clinical trials

Phages are currently being tested as dietary supplements and therapeutic agents promoting a balanced and anti-inflammatory overall gut ecosystem in human subjects. In this section, we are only covering regulated clinical trials intended for improving gut health. Of note, other phage mixtures such as intestiphage have been successfully used in the past two decades to prevent and treat intestinal problems caused by various pathogens (Kutateladze and Adamia, 2008). Two studies targeting *E. coli* followed healthy individuals and acute bacterial diarrhea children examining the effect of phage cocktails on the gut microbiota, intestinal inflammation, and improvement of clinical symptoms (Sarker et al., 2016; Febvre et al., 2019; Gindin et al., 2019; Grubb et al., 2020). In a first study, following a double-blinded, placebo-controlled crossover trial, healthy individuals with self-reported gastrointestinal issues (58% with detectable levels of *E. coli* prior to treatment start) consumed phages for 28 days and collected stool and blood samples to observe any changes in gut inflammation and microbiota. It was shown that *E. coli* abundance decreased in feces with phage consumption without the disruption of the overall bacterial gut community, except for an increase of beneficial fermentative butyrate-producing *Eubacterium* and a decrease of pro-inflammatory *Clostridium perfringens*. In addition, authors observed a significant decrease in circulating interleukin-4 when phages were consumed. Taken altogether, this suggest that phage addition was effective against *E. coli* and aided in maintaining a healthy gut environment (Febvre et al., 2019; Gindin et al., 2019). Phage addition as a combination therapy to probiotic intake could extend the improvement of gut function and reduction of GI distress. The phage cocktail was added to the probiotic *Bifidobacterium animalis* and given to healthy participants for 28 days in a randomized, parallel-arm, double-blind, placebo-controlled trial. Compared to individuals consuming probiotics only, those consuming probiotics and phages showed improvement of GI inflammation and colon pain as well as an increased load of beneficial taxa such as *Lactobacillus* and short-chain fatty acid-producing microbial taxa (Grubb et al., 2020).

Federici et al. tested the viability of the orally administered anti-*K. pneumoniae* phage cocktail in healthy adult volunteers in a phase 1 clinical trial. The phage combination or the vehicle were orally administered to 18 adults, twice a day for 3 days at a final concentration of 10^10^ PFU/dose. Their findings showed phage viability, tolerability, persistence, and safety in the lower gut (Federici et al., 2022). Another research group focused on improving acute bacterial diarrhea caused by Enterotoxigenic *E. coli* in children from Bangladesh (all males, aged 6 to 24 months). The oral phage preparations that were given to subjects did not improve diarrhea outcomes compared to standard care. Oral coliphages failed to amplify in the gut and were excreted in the feces possibly due to low phage and *E. coli* concentrations with overgrowth of *Streptococcus gallolyticus* and *Streptococcus salivarius* (Sarker et al., 2016). Of note, no adverse events attributable to oral phage application were observed in any study and genome sequencing of the phages did not identify any virulence factors. Table 1 summarizes the available phage-based interventional clinical trials and their specific patient population, medical condition, treatment, and outcomes, if any. Overall, there is a need for additional studies focused on the *in vivo* phage-bacterium-host interaction that could explain phage behavior in the ecosystem, optimize phage efficacy against bacterial targets, and improve clinical outcomes.

**Table 1:** Summary of interventional clinical trials investigating bacteriophages and GI diseases.

Title of the study	**Trial status and type**	**Target population**	**Number of participants**	**Trial location**	**Bacterial target**	**Phage cocktail used**	**Therapy**	**Outcomes**	**NCT#**
PHAGE Study: Bacteriophages as Novel Prebiotics	Completed, randomized, double-blind, placebo-controlled crossover trial	Healthy adults with self-reported gastrointestinal distress	43	Colorado State University, USA	*Escherichia coli* and gut bacteriome	PreforPro	1 capsule containing rice maltodextrin and a mixture of 4 bacteriophages or placebo (rice maltodextrin only) consumed 1x daily for 28 days	In phage group: *E. coli* loads decreased in feces No disruption of the bacteriome increase of beneficial *Eubacterium*, decrease of pro-inflammatory *Clostridium perfringens.*	NCT03269617
BacterioPHAGE for Gastrointestinal Health 2 Study (PHAGE2)	Completed, Randomized, double-blinded, placebo controlled, parallel arm	Mild gastrointestinal symptoms in healthy adults	93	Colorado State University, USA	*E. coli* and gut bacteriome	PreforPro as dietary supplement	15 mg capsule containing 10^9^ CFU *Bifidobacterium animals lactis* + 10^6^ PFU PreforPro or *B. animals* alone or placebo with as a filler material, taken orally daily for a period of 4 weeks	In phage+probiotic group: improvement of GI inflammation and colon pain increased load of beneficial taxa and short-chain fatty acid-producing microbial taxa	NCT04511221
A Study to Evaluate the Safety, Tolerability, and Fecal Pharmacokinetics of BX002-A in Healthy Adults	Completed, randomized, single-blinded, placebo-controlled trial	Healthy adults	18	France, Germany, Israel, USA	*Klebsiella pneumoniae*	2-phage cocktail (MCoc5c and 1.2–3 s)	Orally administered 2.8×10^10^ PFU/dose, twice a day for 3 days or placebo Subjects given oral esomeprazole, 40 mg once a day from day-3 to day 3	In subjects receiving phages: -high levels of phages remained detectable in the lower gut. -the two-phage preparation was well-tolerated and safe -no phage treatment-related side effects noted	NCT04737876
Antibacterial Treatment Against Diarrhea in Oral Rehydration Solution	Terminated, Randomized, Double Blind Placebo-controlled Study	6–24 months children, male only	120	Bangladesh	Enterotoxigenic *E. coli* Induced Diarrhea	T4 phage cocktail or Russian anti-*E. coli* phage cocktail (Microgen)	Orally-fed 10^6^ PFU T4 phages or Russian anti-*E. coli* phage cocktail up to 5 days	Phage intake did not improve diarrhea outcomes compared to standard care phage-susceptible *E. coli* colonies appeared in the stool overgrowth of certain *Streptococcus* strains	NCT00937274
Safety and Efficacy of EcoActive on Intestinal Adherent Invasive *E. Coli* in Patients with Inactive Crohn’s Disease, A Phase 1/2a Double-Blind, Randomized, Placebo-controlled trial	Recruiting, Phase 1/2a Double-Blind, Randomized, Placebo-Controlled Trial	Adults with Inactive Crohn’s Disease	30	Intralytix, Inc., Maryland, USA	Adherent Invasive *E. coli*	EcoActive	1 ml of bacteriophage preparation or placebo (saline) given orally twice a day for 15 days	-	NCT03808103
Safety and Efficacy of the Bacteriophage Preparation, ShigActive™, in a Human Experimental Model of Shigellosis	Not yet recruiting, Randomized Intervention	healthy adults (phase 1), and healthy adults after a challenge with Shigella (phase 2a)	52	Intralytix, Inc., Maryland USA	Shigella	ShigActive	1 ml of placebo or phage preparation given orally three times a day for 7 days (Phase 1) or 6 days (Phase 2a)	-	NCT05182749
PrePhage - Faecal Bacteriophage Transfer for Enhanced Gastrointestinal Tract Maturation in Preterm Infants	Not yet recruiting, Randomized trial	Preterm Infants	20	Rigshospitale, Copenhagen, Denmark	Prevention of necrotizing enterocolitis	Fecal Filtrate Transfer, PrePhage	Treatment with fecal filtrate transfer in saline solution administered by nasogastric tube, within 24 h after birth and the following 3 days, in total 4 donations	-	NCT05272579

NCT; National Clinical Trial number.

## The phageome

### The gut phageome ecology

Phages colonize all niches of the body, including the skin, oral and nasal cavities, lungs, gut, genital tract, and urinary tract (Barr, 2017; Divya Ganeshan and Hosseinidoust, 2019). The gut phage population, or gut phageome is a substantial part of the gut with an estimate of 10^12^ total phage within the colon of an average adult human (Clokie et al., 2011; Barr, 2017; Nguyen et al., 2017). It is believed to play a major role in shaping the intestinal ecosystem by modulating the bacteriome diversity through a lytic lifestyle and facilitating horizontal gene transfer while integrated as a prophage (Duerkop et al., 2012; Hsu et al., 2019; Shkoporov et al., 2019; Gutierrez and Domingo-Calap, 2020). Moreover, phages can physically interact with the mucosal surfaces, bypass epithelial cell layers, disseminate throughout the body and may manipulate the immune system (Barr, 2017; Nguyen et al., 2017). In fact, the phageome has been shown to impact immunity directly by modulating the innate immunity *via* phagocytosis and cytokine responses and impacting the adaptive immunity *via* effects on antibody production, enabling anti-inflammatory properties (Gorski and Weber-Dabrowska, 2005; Gorski et al., 2018; Van Belleghem et al., 2018). In particular, it was shown to play a role in lowering the risk of clinical GI complications in stem cell transplant recipients (HCT) (Gorski et al., 2018).

Interestingly, there is a substantial variation of intestinal phageome between individuals, even when those individuals have similar bacterial community structures (Van Belleghem et al., 2018). In fact, when comparing the bacterial and viral population in CRC patients, the CRC bacterial fecal signature tends to be primarily dominated by a limited number of key drivers such as *Fusobacterium*, whereas the phageome signature appears to be more diverse (Hannigan et al., 2018a; Handley and Devkota, 2019). Longitudinal metagenomic studies have shown that the human gut phageome is highly personalized consisting of a (1) healthy core that is highly prevalent, persistent, and individual discriminatory composed primarily of lytic *Caudovirales* phages (crAss-like, *Siphoviridae*, *Myoviridae*, *Podoviridae*), and members of the family *Microviridae* which are believed to infect the majority of the bacterial microbiota and (2) a non-persistent transiently phageome that is less stable, less abundant, and less individualized (Reyes et al., 2010; Barr, 2017; Draper et al., 2018; Moreno-Gallego et al., 2019). The core phageome has been shown to have high levels of inter-personal diversity and within-person conservation for periods of at least 1 year and up to 26 months (Minot et al., 2011; Shkoporov et al., 2019).

As the core phageome is highly prevalent, it was postulated that it may play a key role in gut health and cancer progression (Hannigan et al., 2018b). In fact, these core phages were found to be significantly decreased in individuals with gastrointestinal disease such as UC and CD, individuals with malnutrition, obesity, and GI graft versus host disease (GI GvHD) (Norman et al., 2015; Reyes et al., 2015; Kim and Bae, 2016; Barr, 2017; Legoff et al., 2017; Clooney et al., 2019). Indeed, a decreased abundance of *Clostridiales* phages and a higher abundance of *Streptococcus* phages were observed in mice models of colitis as well as human IBD patients, making them potential IBD markers (Norman et al., 2015; Duerkop et al., 2018). Additionally, although *Bacteroidetes* abundances decreased during dysbiosis, *Bacteroidetes* phages abundance increased. This could be the result of prophage excision from the Bacteroidetes chromosomes caused by environmental stresses such as host inflammatory products and nutrient availability (Turnbaugh et al., 2006; Duerkop et al., 2018). Furthermore, *Enterobacteria* phages and *Escherichia* phages were more abundant in the mucosa of 167 UC subjects than healthy controls, independently of the geographical region. Enrichment of *Caudovirales* bacteriophages along with decrease in diversity was observed in UC subjects. Additionally, patients with UC showed an increase in bacterial virulence and fitness functions acquired by phages and a loss of viral-bacterial correlations in the mucosa (Zuo et al., 2019). Another GI disorder where the gut microbiome is considered an important factor in pathogenesis is irritable bowel syndrome (IBS). It is characterized by intermittent abdominal pain and altered bowel habits (Mihindukulasuriya et al., 2021). One study compared the phageome of diarrhea-predominant IBS and constipation-predominant IBS compared to healthy controls. The authors found a decrease abundance of the *Microviridae* targeting *Chlamydia trachomatis* and an increase of *Rhodococcus Siphoviridae* in diarrhea-predominant IBS compared to constipation-predominant IBS (Mihindukulasuriya et al., 2021). Additionally, the composition of the gut phageome has been shown to be altered at the different stages of CRC progression with a high abundance of *Streptococcus* phages at the early-stage CRC and *Parabacteroides* phages exponentially increasing at the late CRC stages (Nakatsu et al., 2018; Kannen et al., 2019). The analysis of the phageome and bacteriome of 60 CRC patients in a cross-section study showed that many phages belonging to the *Siphoviridae* and the *Myoviridae* families were associated with cancer progression compared to healthy controls. Additionally, no correlation was observed between the bacterial and phage relative abundance ascertaining that the phage signals do not represent reflections of the bacteria (Hannigan et al., 2018b). When observing the phageome of 44 HCT recipients who experience GI GvHD, a study showed that a decreased phage richness and a higher abundance of *Microviridae* specifically during the first few weeks post-HCT is predictive of GI GvHD initiation (Legoff et al., 2017). It is thought that the dynamics between the enteric phageome and the bacteriome changes during a pathological condition compared to a healthy state whereby the phage multiplication results in the loss of bacteriome balance and the emergence of opportunistic bacteria such as *F. nucleatum* and others leading to gut inflammation and cancer progression (Norman et al., 2015; Handley and Devkota, 2019).

### Filtered fecal transplantation

Fecal microbial transplantation (FMT) is the transfer of fecal matter from a donor to the recipient’s intestinal tract and is used to directly modify the gut microbial composition of the recipient and improve gut health (Waller et al., 2022). The clinical value of FMT has been clearly demonstrated in patients with recurrent *Clostridioides difficile* infection (CDI), with beneficial outcomes surpassing standard of care antibiotic regimens (Hvas et al., 2019). Interestingly, the use of filtered fecal transplantation (FFT), where FMT preparations are sterile-filtered, has shown similar outcomes in 5 immunocompromised patients with *C. difficile*, sufficiently restoring their normal stool habits and eliminating symptoms (Ott et al., 2017). This could suggest that the filtered content of FMT, mostly composed of phages, could have a beneficial impact on the gut microbiome balance and the improvement of clinical outcomes (Ott et al., 2017). FFT has also been employed recently in treating gastrointestinal diseases including IBS and resulted in improvement of outcomes (Ott et al., 2017; Lin et al., 2019; Draper et al., 2020; Rasmussen et al., 2020b; Brunse et al., 2022). Successful FMT was associated with significant abundance of phages belonging to the *Caudovirales* order in patients with UC and GvHD, emphasizing the potential role of phages in the efficacy of FMT (Chehoud et al., 2016; Gorski et al., 2018; Zhang et al., 2021a). Similarly, a study compared the effect of FFT to FMT in modifying the outcomes of SIBO. Using fecal filtrates was associated with a reduced bacterial density in mice suffering from SIBO, subjected to a 30-day high-fat diet (Lin et al., 2019) and alleviated type-2 diabetes in mice (Rasmussen et al., 2020b). Of note, authors observed that FFT alone produced the same outcome than FMT, demonstrating the potential role of FFT in the modulation of the gut microbiome (Lin et al., 2019). Additionally, another study showed the beneficial effect of FFT on preventing necrotizing enterocolitis (NEC) initiation (Brunse et al., 2022). Necrotizing enterocolitis is a lethal inflammatory and necrotic bowel disease mostly affecting preterm infants. It was shown that infants with NEC have increased *Proteobacteria* and reduced *Bacteroides* abundance with a lack of obligate anaerobes and overabundance of single facultative anaerobes (Brunse et al., 2022). Using preterm piglets as a relevant model, this study showed that orally-administered FFT but not FMT successfully prevented NEC by reducing intestinal permeability, decreasing *Proteobacteria* abundance in the mucosa, and increasing viral diversity with no recognizable side effects (Brunse et al., 2022). Interestingly, virome analyses showed an increase in the abundance of pathogenic eukaryotic viruses such as *Herpesviridae* in the FMT-treated piglets whereas *Caudovirales* phages were dominating the gut of FFT-treated piglets (Brunse et al., 2022; Figure 1). To associate the efficacy of FFT to phages rather than other nanoparticles (Rasmussen et al., 2020a), Draper et al. investigated the role of autochthonous FFT in the bacteriome recovering post-antibiotic treatment in mice comparing FFT to heat-treated and nuclease-treated FFT, killing and inactivating the phages in the latter strategy. Metagenomic sequencing showed that the bacteriome of the FFT-treated mice had a closer resemblance to the pre-antibiotic treatment compared to the bacteriome of mice receiving non-viable phages (heat and nuclease-treated FFT). Indeed, phages persisted in mice receiving FFT highlighting the capacity of this intervention in restoring the gut microbiota and preventing bacterial infections post-antibiotic treatment (Draper et al., 2020).

## Role of temperate phages

### Vectors for horizontal gene transfer

Temperate phages dominate the gut microbiome (Reyes et al., 2010; Minot et al., 2011; Nguyen et al., 2017; Kannen et al., 2019). The genomes of temperate phages usually contain the integrase gene that aids in the integration of the phage into the bacterial genome as prophages. Environmental stressors such as UV radiation, antibiotics, western diet (high-fat, high-sugar, and low-fiber intake), and others can cause the induction of these prophages, activating phage replication and the release of the phage progeny that might harbor virulence and antibiotic-resistance genes (Lin and Lin, 2019). These released temperate phages can potentially integrate into other bacterial cells contributing to the spread of novel bacterial virulence and pathogenic traits *via* horizontal gene transfer (Reyes et al., 2010; Minot et al., 2011; Nguyen et al., 2017; El Haddad et al., 2018). Indeed, an inflamed gut is associated with a loss of phage diversity and an increased abundance of prophage inductions triggering the adhesion, colonization, and invasion of bacterial pathogens. In the case of shiga toxin-producing *E. coli*, antibiotic intake initiates an SOS response in bacteria that in turn, activates shiga toxin synthesis and secretion *via* prophage induction causing diarrhea and sometimes, fatal hemorrhagic colitis (Brüssow et al., 2004; Lin and Lin, 2019). Furthermore, temperate phages can contribute to disease pathogenicity in IBD patients. Through whole-metagenome sequencing generated by the IBD Multiomics Database project, Nishiyama et al. associated phages to their hosts *via* genome comparisons and found that temperate phage abundance varies between IBD versus non-IBD patients (Nishiyama et al., 2020). More specifically, when comparing active UC patients to non-IBD patients, there was a decrease in the concentration of beneficial bacteria, *Bacteroides uniformis* and *Bacteroides thetaiotaomicron*, and an abundance of temperate phage infecting those bacterial hosts in UC patients. Of note, *B. uniformis* and *B. thetaiotaomicron* were both shown to be assets in gut homeostasis by decreasing the levels of pro-inflammatory interleukins and maintaining the integrity of the epithelial barrier, improving colon damages in IBD subjects (Kuhn et al., 2018; Hiippala et al., 2020; Nishiyama et al., 2020).

Conversely, the presence of prophages could also be linked to the protection of the intestinal epithelium against colitis development. Indeed, Nishio et al. showed that the presence of prophage phiEG37k in the genome of *Enterococcus gallinarum*, a bacterium found at increased abundance in mice with colitis, produced more Mucin 2 that protects the intestinal epithelium compared to its absence (Nishio et al., 2021).

### Engineered phages for strain-specific depletion

With the potential emergence of phage resistant bacteria and the narrow host range of natural phages, alternative methods for pathogen depletion involving phage engineering recently started to develop (Kannen et al., 2019; Selle et al., 2020; Lenneman et al., 2021). Although temperate phages do not serve as good candidates for bacterial lysis in the context of therapy, their ability to integrate into the bacterial genomes as prophages and advances in synthetic biology opened the door to designer phages, engineered phages that possess enhanced properties over the wild type phages. Those properties involve the broadening of the phage host range, increasing biofilm degradation, eliminating lysogeny, and adding payload genes as a mean of increasing antibacterial therapy, suppressing bacterial resistance to phage infection, and preventing antibiotic-resistant infections *in vivo* (Selle et al., 2020; Hsu et al., 2020a; Lam et al., 2021; Lenneman et al., 2021).

The host range and specificity can be altered or reprogrammed by modifying the phage receptor binding modules located at the distal end of the phage baseplate or the tip of the tail fibers which are used to recognize the bacterial surface receptors (Lenneman et al., 2021; Khan Mirzaei and Deng, 2022). Synthetic biologists are genetically engineering these modules either by gene replacements or point mutations. Through site-directed mutagenesis and following the identification of the host-range-determining regions in the T3 phage, a T7-like *E. coli* phage, its receptor binding protein was engineered to generate phagebodies altering their host range, suppressing bacterial growth, and preventing bacterial resistance emergence *in vitro* and *in vivo* (Yehl et al., 2019; Khan Mirzaei and Deng, 2022). Another study focused on the expansion of the host range of phage T7 by constructing hybrid phage particles displaying different phage tails and fiber proteins that can transduce DNA into several novel phage-restrictive bacterial genera other than the target *E. coli* such as *Klebsiella, Salmonella, Shigella*, and *Enterobacter* (Yosef et al., 2017; Khan Mirzaei and Deng, 2022).

**Figure 2:**
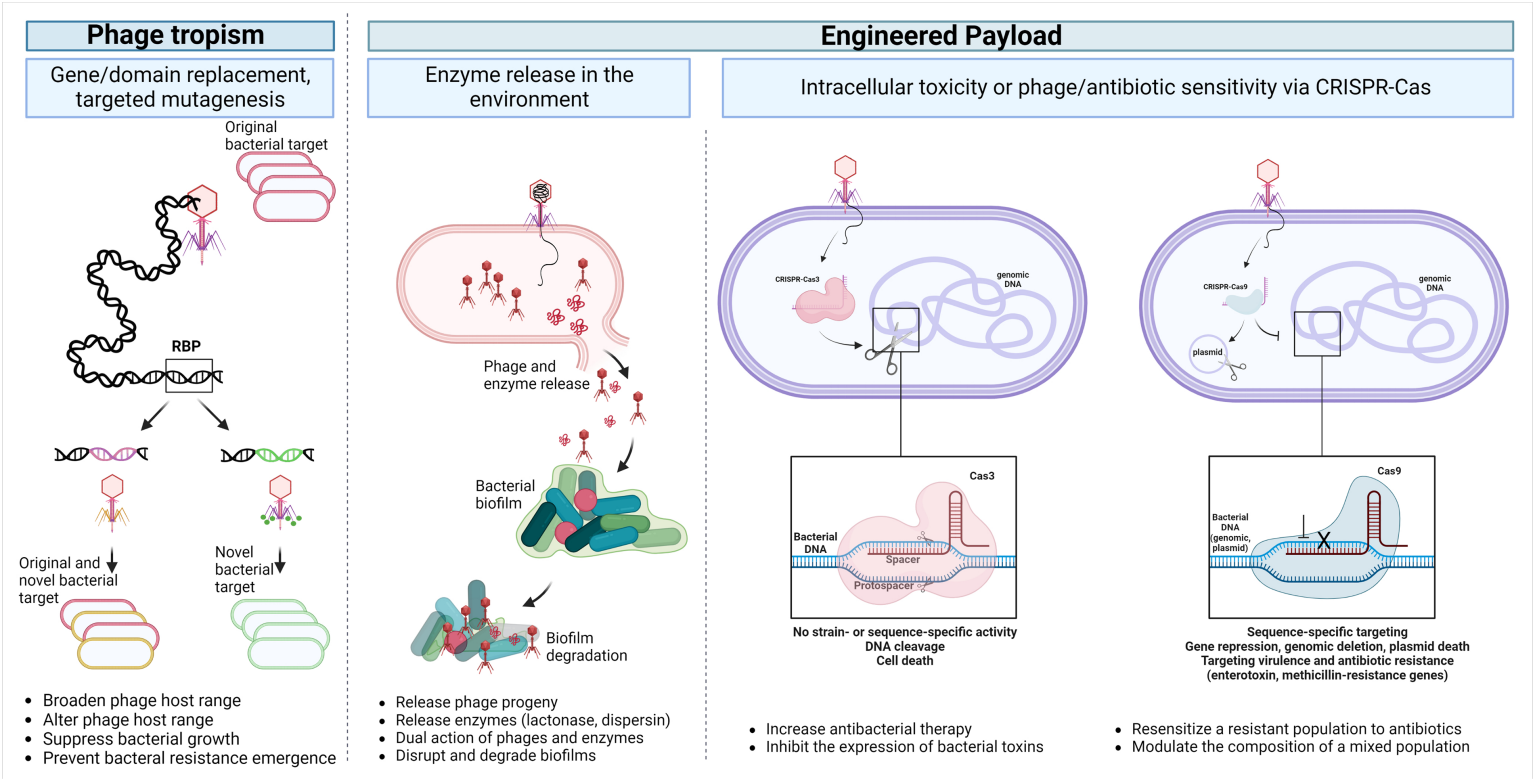
Temperate phage applications. The phage host range can be broadened, modified, or even reprogrammed into a strictly lytic phage by altering the receptor binding modules through point mutations, domain replacement or full gene replacement (Phage tropism). Additionally, genetic payloads can be engineered into the phage genome to enhance or modulate the antimicrobial activity of phages enabling the release of biofilm and capsule depolymerase, quorum-quenching enzymes, cell wall hydrolases, as well as a programmable Cas nuclease toxin. These payloads can selectively degrade biofilms and remove any resistant or virulent bacterial sub-population (Engineered payload). Abbreviations: RBP, receptor-binding protein; CRISPR, Clustered, Regularly Interspaced, Short Palindromic repeats. Created with BioRender.com.

Figure 2 shows different means to modify phages to target new hosts or deliver payloads for specific biological outcomes.

Phage efficacy can also be enhanced through the production of genetic payloads to overcome bacterial anti-phage mechanisms and enhance phage efficacy by targeting and modifying biofilm depolymerases, quorum-quenching enzymes, and cell wall hydrolases (Lenneman et al., 2021; Khan Mirzaei and Deng, 2022). Lu et al. engineered T7 *E. coli* phage to express the dispersin B during infection, releasing this enzyme into the environment upon cell lysis. The dispersin B hydrolyzes the adhesin needed for biofilm formation and structural rigidity in *E. coli.* Authors found improved efficacy in disrupting the biofilm formed by *E. coli* when using dispersin-expressing T7 compared to the T7 wild-type (Lu and Collins, 2007). Similarly, another study showed that the engineering of a T7 phage to express the lactonase enzyme resulted in the degradation of necessary chemical molecules acyl homoserine lactones and the inhibition of *Pseudomonas aeruginosa* and *E. coli* biofilm formation (Pei and Lamas-Samanamud, 2014). Since bacterial biofilm formation is associated with CRC and could promote tumorigenesis in preclinical models (Dejea et al., 2014; Tomkovich et al., 2019), these findings may have chemo-preventive implication. Furthermore, Kilcher et al. used cell wall deficient *Listeria monocytogenes* L-form bacteria to produce synthetic virulent phages upgrading the temperate properties of the phages to lytic and incorporating “enzybiotics” payload in their genomes to target phage-resistant bacteria (Kilcher et al., 2018).

Another example of payload engineering system is the clustered, regularly interspaced, short palindromic repeats and the Cas RNA-guided nuclease (CRISPR-Cas). The phages, or phagemids, can be engineered to deliver a programmable sequence-specific antimicrobial, i.e., Cas nuclease toxin, that can specifically cleave the DNA of the bacterial host or re-sensitizing them to antibiotics by targeting a specific sequence in the chromosome or plasmids carrying virulence or antibiotic resistance genes (Bikard et al., 2014; Gomaa et al., 2014; Selle et al., 2020). Type I (Cas 3 nuclease) and type II (Cas9 nuclease) CRISPR-Cas systems have been used in studies to target different pathogens. The CRISPR-Cas3 system is predominant in prokaryotes that targets and degrades chromosomal DNA resulting in bacterial death but does not have a strain or sequence-dependent nucleolytic activity (Gomaa et al., 2014; Selle et al., 2020). The antimicrobial properties of CRISPR-Cas3 were explored in *C. difficile* where the temperate phage ΦCD24-2 was engineered to enhance its lytic activity and include desirable payload (host-targeting toxin) leading to bacterial genome degradation and the improved reduction of lysogeny *in vitro* and in a *C. difficile* mouse infection model (Selle et al., 2020; Lenneman et al., 2021). The CRISPR-Cas9 system has been used against enterohemorrhagic *E. coli via* sequence-specific targeting. The delivery of this system *via* a temperate phage enabled the modulation of the bacterial population of larvae *Galleria mellonella* and significantly improved their overall survival (Citorik et al., 2014). In another study, *E. coli* was targeted using the single-stranded DNA filamentous phage M13 without causing cell lysis. Using engineered Cas-9 delivering M13 phagemids, one of the 2 competitive *E. coli* colonizers was selectively depleted within the mouse GI tract (Lam et al., 2021). In addition, as a non-invasive and minimally disruptive method, engineered temperate phage λ has been successfully used to precisely repress Shiga toxin expression *in vitro* and in the mammalian gut as well as repress another targeted *E. coli* gene with a single oral dose *via* a programmable nuclease-deactivated Cas9 system (Hsu et al., 2020a, 2020b).

## Discussion

Phage research has been investigated as a therapeutic agent in the past few years as either the wild type or engineered version. Although several promising studies have shown the efficacy of phage use *in vivo*, several main points should still be investigated further before this technology is employed in humans. Indeed, it was speculated that phage-susceptible bacteria may be present and disseminate in phage-inaccessible sites in the murine mucosa contributing to the co-existence of phages and their phage-susceptible bacteria and the possible low efficacy of orally administered phages (Lourenço et al., 2020). Hence, the viability and storage of phages or phage products is still scarce. Microencapsulation of phages has been used to stabilize the phage and allow their efficient release in the intestinal environment. Yin et al. showed that phages packed in sodium alginate/calcium chloride microcapsules were more resistant in the simulated intestinal fluid and were more efficient in killing *E. coli* in rats compared to free phages (Yin et al., 2021). Moreover, in an effort to optimize the oral delivery of an engineered phage system in the mouse gut, Hsu et al. developed an aqueous-based encapsulation formulation (alginate beads coated in polyelectrolyte multilayer films) with a microbiota-based release mechanism that enables release when entering the bacterial-dense large intestine (Hsu et al., 2020a). Other challenges in phage research include the isolation of phages from clinical samples, library preparation and sequencing strategies. Most studies focusing on phages only isolate double stranded viral DNA and exclude RNA and single-stranded DNA viruses, thus potential important ecological interaction are ignored (Handley and Devkota, 2019). Additionally, 95% of the viral sequences are classified as “unknown” thus preventing researchers to complete phageome characterization, the interaction phage-bacteria-host, and the phageome association with disease (Handley and Devkota, 2019). In addition, as more metagenomic studies are revealing increased genomic diversity, new taxonomic rankings are being recently put in place, abolishing the order *Caudovirales* and families *Myoviridae*, *Podoviridae*, and *Siphoviridae* that were based on phage morphology types. This review has quoted the taxonomy mentioned in the different citations in hopes that the new classifications will be implemented and unified in future studies (Turner et al., 2021). Moreover, challenges in limited culture-based method render some bacterial strains and their respective phages unculturable and are more difficult to isolate, particularly phages and their anaerobic hosts (Khan Mirzaei and Deng, 2022). On another note, the administration route constitutes another challenge for phage use. Selecting a specific route could potentially alter the clinical outcomes. It was shown that orally delivered phages were safer and more effective than intraperitoneally administered phages in inhibiting tumorigenesis in pre-clinical models (Dabrowska et al., 2005). Furthermore, the intake of dietary compounds such as polyphenols, teas, and sweeteners can have the potential to impact the physiology of phages by either inhibiting phage activity or promoting phage responses (Marongiu et al., 2021). Thus, diet is an essential element in the involvement of phages in the combat against GI diseases and cancer progression (Marongiu et al., 2021).

Bacteria coexist with hundreds of other species in the gut despite diet changes, phage infections and other perturbations. Indeed, these bacteria have evolved strategies to persist in their environment and evade phages such as the modification of surface receptors at high frequencies or phase variation, the restriction-modification systems and the CRISPR loci or adaptive immunity of bacteria (Labrie et al., 2010; Bikard and Marraffini, 2012). For example, gut symbionts such as *Bacteroides thetaiotaomicron* produce multiple phase-variable capsular polysaccharides and other lipoproteins generating phenotypic heterogeneity and altering host tropism resulting in bacterial survival and escape from phage predation (Porter et al., 2020).

Lastly, phages may have a role in either immune homeostasis and the aggravation of the immune responses *in vivo* (Gorski et al., 2018; Gogokhia et al., 2019). Phages alone can downregulate the levels of pro-inflammatory cytokines and chemokines, such as tumor necrosis factor (TNF-alpha), interleukin 1 (IL-1), Toll-like receptor 4 (TLR4), reactive oxygen species (ROS), CXCL1, and CXCL5. They can also induce anti-inflammatory ILs (ILs 4, 10, ILRa) and suppressor of cytokine signaling 3 (SOCS3), with subsequent dampening of acute and chronic GI GvHD (Van Belleghem et al., 2017; Gorski et al., 2018). Interestingly, a prophage integrated in the bacterium *Enterococcus hirae* enhanced immunotherapy-mediated anti-tumor response through an MHC-class I epitope encoded in the tail-length tape measure protein (TMP) of the bacteriophage (Fluckiger et al., 2020). This suggests that component of bacteriophages could be utilized as immunostimulatory tools. However, some phages may encode ankyrins that can facilitate bacteria evasion and survival in different ecosystems including the gut by decreasing macrophage phagocytosis rates and reducing expression of pro-inflammatory cytokines (Jahn et al., 2019). Similarly, treatment with phages have been also shown to trigger immune cell expansion with the stimulation of interferon gamma, exacerbated gut inflammation in a mouse model of colitis (Gogokhia et al., 2019). This challenge could be overcome by genetically engineering the immunogenic phage proteins to lower inflammation and preventing the neutralization of phages by the immune system, even though genetic engineering of phages is still in its infancy (Hodyra-Stefaniak et al., 2019; Khan Mirzaei and Deng, 2022).

## Conclusion and perspectives

Biological strategies involving the use of phages, natural or engineered, constitute a novel and non-invasive area of investigation aimed at the restoration of intestinal bacteriome balance, the decrease of infection and inflammation, and the prevention of cancer progression. Research on phages has shown great potential for their use and their safety in mammalian hosts, however much work is still needed to understand the dynamics between phages, bacterial host, bacteriome, and the mammalian host stratified by type of phages (engineered versus wild type phage) and different cohorts of diseases and stages of diseases.

## Author contributions

LEH and JFM wrote the manuscript. LEH and CJ contributed to reviewing and editing the manuscript. All authors contributed to the article and approved the submitted version.

## Funding

CJ was supported by NIH DK073338, NIH CA264927 and AI166096. LEH was supported by NIH AI166096 and NIH CA264927. The funders had no role in study design, data collection and analysis, decision to publish, or preparation of the manuscript.

## Conflict of interest

The authors declare that the research was conducted in the absence of any commercial or financial relationships that could be construed as a potential conflict of interest.

## Publisher’s note

All claims expressed in this article are solely those of the authors and do not necessarily represent those of their affiliated organizations, or those of the publisher, the editors and the reviewers. Any product that may be evaluated in this article, or claim that may be made by its manufacturer, is not guaranteed or endorsed by the publisher.
